# Heat Stress and Goat Welfare: Adaptation and Production Considerations

**DOI:** 10.3390/ani11041021

**Published:** 2021-04-04

**Authors:** Veerasamy Sejian, Mullakkalparambil V. Silpa, Mini R. Reshma Nair, Chinnasamy Devaraj, Govindan Krishnan, Madiajagan Bagath, Surinder S. Chauhan, Rajendran U. Suganthi, Vinicius F. C. Fonseca, Sven König, John B. Gaughan, Frank R. Dunshea, Raghavendra Bhatta

**Affiliations:** 1 Centre for Climate Resilient Animal Adaptation Studies, ICAR-National Institute of Animal Nutrition and Physiology, Adugodi, Hosur Road, Bangalore 560030, India; mv.silpa@gmail.com (M.V.S.); reshmasarovaram@gmail.com (M.R.R.N.); drcdeva@gmail.com (C.D.); vet.krish@gmail.com (G.K.); bbagath@gmail.com (M.B.); r.umayasuganthi@gmail.com (R.U.S.); ragha0209@yahoo.com (R.B.); 2 Institute of Animal Breeding and Genetics, Justus-Liebig-Universität Gießen, 35390 Gießen, Germany; sven.koenig@agrar.uni-giessen.de; 3 Academy of Climate Change Education and Research, Kerala Agricultural University, Vellanikkara 680656, India; 4 School of Agriculture and Food, Faculty of Veterinary and Agricultural Sciences, The University of Melbourne, Parkville, VIC 3010, Australia; ss.chauhan@unimelb.edu.au (S.S.C.); fdunshea@unimelb.edu.au (F.R.D.); 5 Innovation Group of Biometeorology and Animal Welfare, Animal Science Department, Universidade Federal da Paraíba, Areia 58397-000, Brazil; vinicius.fonseca@academico.ufpb.br; 6 Brain Function Research Group, School of Physiology, Faculty of Health Sciences, University of the Witwatersrand, Parktown 2193, South Africa; 7 School of Agriculture and Food Sciences, The University of Queensland, Gatton, QLD 4343, Australia; j.gaughan@uq.edu.au; 8 Faculty of Biological Sciences, The University of Leeds, Leeds LS2 9JT, UK

**Keywords:** climate, breeding, genetics, goat, heat stress, housing, transportation, welfare

## Abstract

**Simple Summary:**

This review attempts to provide information on the various impacts of heat stress on the production and welfare variables of goats. Goats are predominantly distributed in tropical regions, and hence they are considered a species readily able to survive and produce in these demanding environments. However, the production of goats can be compromised to a degree to survive in harsh environments. Hence, having an in-depth understanding of the various impacts of heat stress on goat production and adaptation may yield important biomarkers to assess welfare in goats. Such efforts may help in the future to breed goats, which could survive and produce optimally under harsh conditions.

**Abstract:**

This review attempted to collate and synthesize information on goat welfare and production constraints during heat stress exposure. Among the farm animals, goats arguably are considered the best-suited animals to survive in tropical climates. Heat stress was found to negatively influence growth, milk and meat production and compromised the immune response, thereby significantly reducing goats’ welfare under extensive conditions and transportation. Although considered extremely adapted to tropical climates, their production can be compromised to cope with heat stress. Therefore, information on goat adaptation and production performance during heat exposure could help assess their welfare. Such information would be valuable as the farming communities are often struggling in their efforts to assess animal welfare, especially in tropical regions. Broadly three aspects must be considered to ensure appropriate welfare in goats, and these include (i) housing and environment; (ii) breeding and genetics and (iii) handling and transport. Apart from these, there are a few other negative welfare factors in goat rearing, which differ across the production system being followed. Such negative practices are predominant in extensive systems and include nutritional stress, limited supply of good quality water, climatic extremes, parasitic infestation and lameness, culminating in low production, reproduction and high mortality rates. Broadly two types of methodologies are available to assess welfare in goats in these systems: (i) animal-based measures include behavioral measurements, health and production records and disease symptoms; (ii) resources based and management-based measures include stocking density, manpower, housing conditions and health plans. Goat welfare could be assessed based on several indicators covering behavioral, physical, physiological and productive responses. The important indicators of goat welfare include agonistic behavior, vocalization, skin temperature, body condition score (BCS), hair coat conditions, rectal temperature, respiration rate, heart rate, sweating, reduced growth, reduced milk production and reduced reproductive efficiency. There are also different approaches available by which the welfare of goats could be assessed, such as naturalistic, functional and subjective approaches. Thus, assessing welfare in goats at every production stage is a prerequisite for ensuring appropriate production in this all-important species to guarantee optimum returns to the marginal and subsistence farmers.

## 1. Introduction

Livestock production is considered the most widely adopted agriculture practice by marginal and subsistence farmers, particularly in the developing part of the world [1]. However, sustaining livestock production has become a challenging task in the changing climate scenario [2]. Beyond the direct consequences, climate change disrupts animal agriculture by reducing both pasture and water availability as well as by increasing the frequency of sudden disease outbreaks [3]. The reduction in pasture availability and shrinking grazing lands has caused a marked reduction in livestock production in recent years. Therefore, these unavoidable adverse effects have focused research efforts towards identifying the most climate-resilient animals across different livestock species.

With the increasing concern in securing the global economic viability, recent research efforts have ascertained goats as the ideal climate animal model due to their better thermo-tolerance [4], drought tolerance [5], ability to survive on limited pastures [6] as well as their disease resistance capacity [7]. In the context of indirect effects of changing climate pertaining to feed and fodder availability, rearing goats is considered more economical than large ruminants.

In terms of numbers, geographic distribution and socioeconomic importance, goats are among the most critical global livestock species [8]. Together with sheep, they were the first ruminants to be domesticated around 11,000 years ago in the fertile crescent. They were subsequently dispersed across the globe, adapting themselves to diverse biophysical and production environments. Over the years of evolution, human intervention or artificial selection for production, reproduction and physical traits derived breeds of goats of temperate regions, which have been utilized in the specialized production systems in Mediterranean countries of Europe. To produce milk, natural selection was the most prominent factor for the evolution of goats dispersed in dryland regions [9]. In contrast with the phenotypic uniformity of temperate breeds, the goat population living under harsh environments has broad diversity traits and a lack of genetic structure, which provides evidence for high plasticity capacity that facilitates their productivity across a wide range of environmental conditions [10].

In seasonal biotopes of arid and semi-arid regions over the world, climate change increased prolonged drought events, and erratic weather has limited the production of large ruminants, such as cattle and buffalos, as opposed to the progressive increase in the rearing of goats [8]. Under such conditions, herders perceive goats as a more resilient animal to cope with multiple stressors, such as heat load, water and feed scarcity, with better skills to cope with bush compared to sheep and cattle [11]. There are many scientific pieces of evidence clarifying the particular traits of goats that help them to cope with environmental challenges in different types of ecosystems, which mostly include: first, the small body size of goats, which allows them to escape more efficiently from the high radiant heat load by using thermally buffered microclimates, as well as, a lower absolute requirement for energy, water, and home range [12,13]. Second, the unique capacity for employing behavioral plasticity and goats’ morphological features imparts them the clear advantage over sheep and cattle to cope with seasonal biotopes with a lack of both feed and water [14,15]. Lastly, when facing low-quality feed, they also are superior to cattle and sheep in their ability to digest dry matter and recycle nitrogen [16]. These physiological, behavioral and morphological advantages in goats make them suitable species to survive in diversified geomorphological conditions.

Although there is sufficient information available to conclude that goats are the more climate-resilient species [4], still their production is not devoid of adverse impacts of climate. There are enough pieces of evidence for the reduced production, reproduction, health variables and energy efficiency due to heat stress in goats [17,18]. However, studies assessing the resilient capacities of different species have delineated goats to have a minimum deviation from their optimum performance efficiency [4,6]. Thus, the promotion of goat production can be considered an important step towards sustainable livestock production in the changing climate scenario.

Therefore, understanding in-depth the heat-stress-associated adversities in goat production and adaptation may help to ensure their welfare. Thus, this review primarily focuses on assessing the welfare of goats during heat stress exposure. The review also aims to provide useful information about different approaches and methodologies in assessing goat welfare.

## 2. Goat as the Future Animal from Food Security Perspectives

Sustaining livestock production under a challenging climate has necessitated the need for identifying an ideal species to cater to the needs of the growing human population. Several studies have identified goats as the go-to species to sustain animal agriculture under changing environmental conditions [19,20,21]. Pioneers in livestock research had identified the potential of goats over other small ruminants to adapt to a wide range of environmental conditions [22]. Goats are opportunistic feeders, and thus the depletion of pasture lands may hardly impose an impact on their dietary requirements. Moreover, the selective feeding behavior of goats help them consume even the poor quality forages, converting the nutrients obtained into high-quality products [23,24]. In addition, goats exhibit a bipedal stance, which helps them access tree leaves, which is considered advantageous to other livestock species [22]. Further, goats have a better feed efficiency than other ruminant species. In addition, goats do not require specialized shelter structures, and they could ideally survive in any location with minimum protection from the weather [22,25]. Additionally, labor availability is another crucial factor for livestock production, but which is considered less of a big constraint in goat production since much of the labor could be done by family members. Indeed, Rokonuzzaman and Islam [26] revealed that 20 to 48% of women were involved in goat rearing.

The world’s population is expected to touch an alarming count of 9.6 billion by 2050. From the food security perspective, animal proteins are considered vital to meet the growing demands of the human population, especially in the developing world. Goats are projected as the ideal climate-adapted animals and are expected to perform better than other species. This projects their pivotal role in meeting the growing humanitarian needs for animal protein by the end of this century. Further, goats are also expected to perform better than other livestock species, particularly given the climate change-associated feed and fodder shortage. Therefore, researchers and policymakers should set priorities in designing appropriate programs to meet the growing human population’s food demands by 2050.

In the context of the anticipated increase in the human population, goats play a vital role in catering to future generations’ nutritional demands through the production of milk and meat [27]. As per the model prepared by Ngambi et al. [28], dairy goats produce approximately 15.2 million tons of milk, comprising 2% of total milk production from the livestock sector. Moreover, goat meat and milk demand have been rising exponentially above other livestock species for their health benefits and therapeutic values [29]. In harmony with this, recent reports suggest that goat enterprises have turned out to be of more commercial value as a result of the marketing preference of goat products all over the world [25]. With their unique ability to convert unconventional feedstuff to high-quality animal products, goats play a crucial role in eradicating poverty during disaster aversion [30]. Thus, having the potential scope to ensure food security serves as an important source of income for poor and marginal farmers around the world.

## 3. Heat Stress and Goat Production

Although goats are considered well adapted to the tropical climate, their adaptive responses significantly hamper their production [2]. Some studies established the impact of elevated ambient temperature on growth [20], milk production [31], meat production [19,32] and immune responses [33] in goats. These authors observed a reduction of 12%, 3–10%, and 4% for growth, milk and meat production, respectively. Goats start experiencing heat when they were exposed to 38 °C and above with the THI of above 75 [2]. Once the goats are exposed to this high temperature, they activate their physiological adaptability in terms of alterations in behavior, physiological responses, blood biochemical and endocrinological responses to regulate their body temperature to maintain homeothermy [21]. These adaptive processes are of energy demanding, and the animals channelize their energy from the productive pathways towards the adaptive pathway from the productive [2,21]. Such behavior of reducing the production to support the life-sustaining activities is the typical characteristic of adapted goat breeds.

The adverse impact of heat stress on growth performance can be attributed to the reduction in feed intake, digestibility and utilization efficiency [34,35]. Though goats have the capacity for adaptation to convert poor quality feeds to products in rangelands, still, if the heat stress prolongs for a longer duration, it can affect their growth performance. Further, Pragna et al. [20] established the severity of heat stress on different growth variables in three indigenous Southern Indian goat breeds. These authors observed a reduction of 11.0%, 8.0% and 6.0% of growth for Osmanabadi, Malabari and Salem Black breeds, respectively. Further, heat stress reduces the daily weight gain that subsequently influences their allometric measurements [36]. This reduction in heat-stress-associated growth variables could be due to the outcome of activation of hypothalamus–pituitary–adrenal axis (HPA) in response to heat stress. The activation of the HPA axis during heat stress directly influences the release of GH, negatively influences growth [35]. However, breed variations were observed for these mechanisms of HPA-axis-oriented heat stress impact on growth [20,36].

Besides being a threat to growth, heat stress acts as an aberrant effect on meat production and the goat’s carcass characteristics. Hashem et al. [32] conducted a study to illustrate the effects of heat stress on Black Bengal goats. They reported a reduction in preferred meat quality characteristics, such as pH, cooking loss, water holding capacity, shear force and color. Further, Archana et al. [19] attributed the increased meat pH in heat-stressed goats to glycogen depletion. Apart from the increase in pH, Archana et al. [19] also noted an increased shear force in the meat of heat-stressed Osmanabadi goats, which significantly hampered the tenderness and juiciness. Further, these authors suggested heat stress also reduces the plasticity of muscle fibers, which in turn could contribute, together with ultimate pH, to the alteration in the quality of goat meat. These authors attributed the negative influence of heat stress on meat quantity and quality to depleted energy reserves in the animals as a result of partitioning energy towards life-sustaining activities during the adaptation process, especially when the heat stress is prolonged for a chronic period [19,32].

Several studies were conducted to establish the influence of heat stress on milk production [31,37], where it was ascertained that milk production was reduced considerably in response to reduced feed intake during heat stress exposure in goats. The lactation curve developed by Wood [38] can be used as an indicator to draw a better understanding of the seasonal influence on milk production in dairy goats. In general, the lactation curve of goats, irrespective of breed, invariably declines during hot summer months [39,40,41]. In addition, keeping milk production a major concern, extensive experiments were conducted to determine the effect of heat stress on milk quality and reported a reduction in milk fat, protein, lactose and total solids content, thereby reducing the ultimate milk quality [37,40]. Such an effect could be due to insufficient energy levels as in heat-stressed animals predominantly, the energy is deviated to maintain life-sustaining activities. However, the effect of such impacts varied accordingly with the genetic potential, lactation stage and nutritional availability of goats during their exposure to heat stress [37,41].

Apart from their effects on production traits, heat stress also influences the goats’ immune responses. Sophia et al. [42] noted that innate immune response, which was considered the first line of defense, was compromised in goats after exposure to heat stress. However, various reports show the inefficiency of goats’ primary innate immunity in response to heat stress [43,44]. With the decline in immunoglobulin release, the adaptive immune system becomes impaired, leading to likely parasitic infestation [45]. In agreement with this, Hirakawa et al. [46] noted that extreme temperatures resulted in a limited synthesis of lymphocytes along with suppression in phagocytic activities of leukocytes in goats. In addition, Yadav et al. [47] reported that heat stress depressed the production of antibodies in goats, particularly the production of IgM and IgG. The TLR2, TLR8, IL10, IL18, TNFα, and IFNβ are considered important inflammatory markers for quantifying the impact of heat stress on the immune system in goats [43].

Taken together, it is apparent that the goat compromises their production to cope with adverse environmental conditions and thus threatens the economy of poor and marginal farmers whose primary livelihood depends on goat rearing. Therefore, it is necessary to focus on implementing welfare measures to sustain goat production during heat stress exposure.

## 4. Physiological Mechanisms of Goats towards Adaptation or Reaction to Heat Stress

Upon prolonged exposure to heat stress, goats elicit specific physiological mechanisms that confer them the potential to tolerate heat stress. As an inevitable mechanism that is established during heat stress, physiological variables are often considered among the most reliable goat welfare measures, regardless of breed. Studies on the effect of heat stress in goats have identified a linear relationship between the temperature–humidity index (THI) and the variables associated with the physiological mechanisms [33]. However, the degree of response may vary with the magnitude of heat stress as well as the goat breed.

Dangi and co-workers [33], in their study with Barbari goats, identified respiration rate (RR) as a practical and reliable measure of heat load. The animals utilize the respiratory evaporating cooling mechanism to expel the excess heat, which culminates with the increase in RR. When compared to a basal respiration rate of 15–30 breaths/min, goats exhibiting a respiratory rate of 40–60, 60–80, 80–120 and more than 200 breaths/min are considered to be exposed to low, medium, high and severe heat stress, respectively. Several studies were conducted on various breeds to validate the utilization of evaporative cooling mechanisms in heat-stressed goats. One such study, conducted on Murciano-Granadina dairy goats [48], witnessed an increase in RR up to 150 breaths/min with the prevailing THI value of 77. Similar heat-stress-associated increases in RR occurred in several other indigenous goat breeds [21,33,49].

Nevertheless, the respiratory evaporating cooling mechanism fails when the ambient temperature increases beyond the threshold level. This failure subsequently results in increased body temperature to aid the release of excess heat load. The increase in rectal temperature (RT) is an alternate used by goats to bring down their body temperature to keep themselves under comfort environment. According to Alam et al. [24], indigenous goats exposed to zero, four and eight hours of heat stress showed a significant difference in their rectal temperatures. Further, similar findings occurred in Osmanabadi, Malabari and Salem Black goat breeds of southern India [21], Barbari goats of Northern India [33], and Brown-Pardo-Sertaneja, Azul, Graúna and Moxotó breeds of Brazil [50,51,52,53].

Adaptation to prolonged heat stress may also be incurred through the cutaneous evaporative cooling mechanism by increasing the peripheral blood flow and thereby increasing the pulse rate (PR). With an increase in the circadian rhythm of heart rate, heat exchange occurs both via sensible as well as insensible means to the immediate surroundings of the animals, thereby making their internal environment more comfortable [21,51,52]. Shilja et al. [54] established the increase in PR with Osmanabadi goats’ exposure to heat stress than their counterparts kept in the shed in more comfortable conditions. Similar findings occurred in Boer, Anglo-Nubiana [55] Savana [56] Saanen [57] and Garfagnina [58] goat breeds.

Additionally, skin being the intermediate between the animal body and the surrounding environment, plays a major role in aiding the adaptation of animals to heat stress. With the increased flow of blood towards the periphery of the skin through cutaneous vasodilation, the exchange of heat occurs from the core body to the surroundings. Al-Tamimi [59] found that Damascus male goat kids had higher skin temperature, and they attributed this abrupt increase to heat stress. Many investigations have been conducted to reveal the thermoregulatory mechanism behind sweating. One such notable study by Baker [60] with Alpine Toggenbergs and Nubian goats claimed that the cutaneous moisture loss was less in dehydrated goats. Later, Niliand and Baker [61] found a decline in skin temperature in heat-stressed goats, and they attributed this to the evaporative heat loss through sweating.

## 5. Welfare Considerations in Goat Production

**Figure 1:**
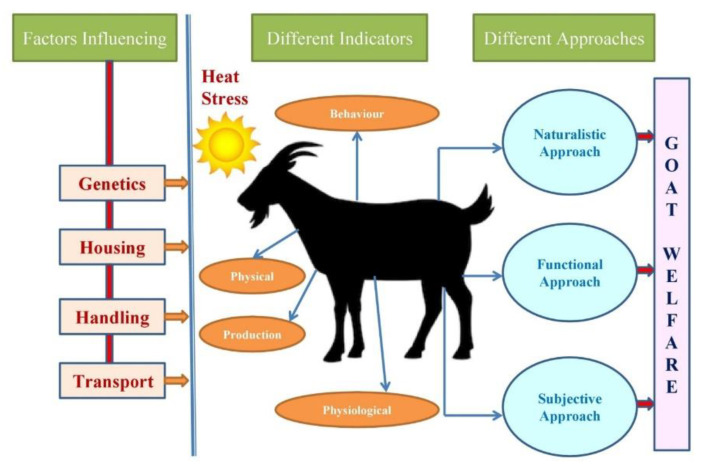
Concept of welfare assessment in goats.

Livestock farming practices have evolved considerably over the decades, with the primary focus to optimize maximum economic returns. Running parallel, the concept of animal welfare has been evolving as once accepted practices are being re-evaluated with the current ethos and environment. With the improving scientific knowledge, researchers are working on including several other aspects of animal behavior and expression to assess their wellbeing. Figure 1 describes the concept associated with assessing welfare in goats. Figure 1 describes the essential factors, such as genetics, housing, handling and transport to influence goat welfare. This figure further highlights the various indicators that pertain to behavioral, physical, physiological and productive functions in heat-stressed goats. In addition, this figure projects three approaches, such as naturalistic, functional and subjective, to assess welfare during heat stress exposure in goats.

### 5.1. Housing and Environment

Recent developments look into the animal’s interaction with the changing environment and also consider their adaptability to such scenarios [62]. Animal housing is one of the prime aspects to be looked at from an animal welfare and production point of view. Sheds providing sufficient spacing and ventilation maintain a hygienic environment within the shed and provide the ultimate comfort to the animals [2]. Goats tend to rest against a wall rather than lying in the center of a pen, which could be an anti-predator strategy adopted by them [63]. In addition to sufficient floor space, the type of floor is also essential as goats prefer solid and dry surfaces [62]. Therefore, it is essential to ensure a clean, dry and adequately ventilated housing environment for the goats, keeping them comfortable and protecting them from disease risk. This ensures the appropriate wellbeing of goats by altering their behavioral variables to assess the housing comfort in these animals.

Haphazard separation and/or introduction of goats into a new flock and repeated separation and reintroduction into the flock is stressful [64]. Therefore, it would be ideal to house the separated or reintroduced goat near the main flock to hear and smell the other goats, reducing the stress on the goat [64]. Other important environmental variables influencing goat production are temperature and humidity. These are vital components that can have deleterious impacts on goat production. Therefore, the housing pattern can protect the animals from these deleterious impacts by providing the optimal microenvironment. The welfare ensured by the housing environment could be reflected in the altered behavior, physiological and metabolic variables.

Broadly, farm animal welfare measures can be categorized into behavioral (abnormal behavior), physiological (heart rate, respiratory rate), health (incidence and prevalence of diseases) and zootechnical (body condition score, mortality and birth rates) categories [53]. The Animal Welfare Indicators for Goats (AWIN Goat) released a protocol to assess the welfare of lactating goats with a goal to generate a holistic picture of the quality of life in animals [65]. As per the protocol, welfare indicators are broadly grouped under the four welfare principles of good feeding, good housing, good health and appropriate behavior. Further, housing principles include three criteria; comfort around resting, thermal comfort and ease of movement. Practicing such on-farm welfare evaluation would ensure maintaining a stable balance between intensive production and goats’ wellbeing.

### 5.2. Breeding and Genetics

Though heat stress severely hampers livestock production, the impact varies among animals both at the species and breed level. It is the genetic potential of an animal that determines its supremacy to withstand climatic adversities [37]. Variations among goats in response to heat stress and adaptive efficiency based on molecular responses in terms of changes in heat shock protein 70 (HSP70), TLR2, TLR8 have been reported [21,66]. Such studies enable the identification and quantification of biomarkers for heat stress, which could further aid in long-term breeding goals towards developing agroecological zone-specific breeds.

Animals exhibit several adaptive responses during heat stress [67]. This area is an emerging field of research aimed at exploring and validating candidate markers for heat stress. In addition to this, the identification of welfare indicators should also be among the top priorities. Body condition score, hair coat condition, resting position, panting score, behavioral responses are a few of the established welfare indicators in goats [63]. However, it is necessary to buildup concrete evidentiary grounds before incorporating the indicators in a valid, reliable, and feasible welfare assessment protocol.

Several improvements have been made in animal breeding policies with the advancements in molecular genetics and their incorporation into animal breeding programs. However, most of these have primarily focused on production traits. With the rising concern of climate change and its impact on livestock production, breeders and policymakers are urging developing thermo-tolerant breeds. Unequivocally, indigenous animals are better adapted to climatic adversities than exotic or crossbred animals expressing better welfare indicators in them. Further, the indigenous breeds show different levels of resilience to climate change [20]. Aleena et al. [21] studied the resilience capacity of three indigenous goat breeds, Osmanabadi, Malabari and Salem Black, during heat stress exposure. On comparing several physiological, behavioral and molecular-level assessments, it was concluded that the Salem Black goats were better adapted to handle heat stress than Osmanabadi and Malabari goats.

The differences in indigenous goats’ adaptive potential to heat stress point towards the importance of screening all the native goat breeds assess their thermo-tolerance potential, and propose superior thermo-tolerant breeds. On comparatively assessing the performance of three different indigenous goat breeds, native to different agroecological zones, during heat stress, Salem Black breed possessed superior adaptive potential than the other two breeds [20]. This study’s exciting finding was that Salem Black goats performed well despite the test location being different from their native zone. Thus, this emphasizes the need to develop agroecological zone-specific breeds, ensuring sustainability in the climate change scenarios.

The genetic basis of thermo-tolerance involves complex processes due to the association of several genes and traits [68]. However, this aspect has been little explored and is now a researchable area for scientists across the globe. Yakubu et al. [69] conducted a study to assess the single nucleotide variations in the MHC class II DRB gene and explored its association with thermo-physiological traits in three major Nigerian goat breeds; West African Dwarf (WAD), Red Sokoto (RS), and Sahel (SH). They identified a total of 14 alleles, of which 7 alleles were significantly associated with heat tolerance. Further, they also reported the SH and RS goats to possess better thermal adaptability to the hot and humid tropical environment prevailing in Nigeria than that of the WAD goats. Likewise, the significance of superoxide dismutases-3 (SOD-3) during heat stress was explored by Khan et al. [70] to identify its polymorphism and correlate the findings with traits associated with heat tolerance in three goat breeds (Black Bengal, Ganjam, Raighar). The authors identified three SNPs in the SOD3 gene that significantly impacted the vital physiological variables and certain plasma biochemical variables, including total proteins, albumin, bilirubin, creatinine, ALT, ALP, glucose and triglycerides. Therefore, the association between the sequence variation in the SOD-3 and the heat stress response variables can also be considered as a vital DNA marker for thermo-tolerance selection.

Apart from the SNP markers, some genes may be genetic markers for the adaptation of goats to heat stress based on their mRNA expression profile. In a study conducted by Shilja et al. [54] to assess the adaptive capability of Osmanabadi goats during summer season-related heat stress and nutritional stress (both individually and as a combined effect), the adrenal HSP70 gene expression was identified to be a potential biomarker. In their study, the HSP70 had a higher mRNA expression in the adrenal glands of goats subjected to combined stresses (combined heat stress and nutritional stress) than that of individual heat or nutritional stress groups. The authors stated that the increased expression of HSP70 would be due to the hyperactivity of the adrenal cortex so as to synthesize cortisol, which again is a vital stress marker. Additionally, Angel et al. [71] established growth hormone (GH), growth hormone receptor (GHR), insulin-like growth factor-1 (IGF-1), leptin (LEP) and leptin receptor (LEPR) genes to be the ideal biomarkers reflecting the growth potential in Malabari goats subjected to heat stress. All the above-mentioned genes significantly reduced their mRNA expression in goats exposed to heat stress.

Heat stress also has severe implications on an animals’ immune response, rendering them susceptible to deadly pathogens. In a study conducted in Malabari goats during heat stress, interleukin 18 (IL-18), tumor necrosis factor-α (TNF-α), interferon-β (IFN-β), and IFN-γ were reliable immunological markers that aid in assessing heat stress-mediated immune response alterations [72]. All these genes’ mRNA expression was significantly downregulated during heat stress in goats. Similarly, Madhusoodhan et al. [66] also reported a significant reduction in hepatic IL-2, IL-6, IL-18, TNF-α and IFN-β mRNA expression in heat-stressed Salem Black goats. They reported that these genes could serve as indicators for heat stress in this breed.

### 5.3. Handling and Transport

The environmental factors, transportation logistics, animals’ management, and handling of individual animals before and after transportation have an adverse impact on animal welfare and health status [73]. The indicators of welfare and stress during transport are assessed in terms of huddling behavior, respiration rate, neutrophil, eosinophil, and blood electrolytes and cortisol concentration to predict the degree of stress. Prolonged livestock species’ transportation causes complex pathophysiological changes, such as increased hemoglobin, packed cell volume, neutrophils and eosinophils, decreased electrolytes and increased cortisol concentration brought by handling, loading, transportation, vehicle movements, and adverse effects of weather variables [74]. The animals on the vehicle experience numerous stressors and feed and water deprivation that elicit complex, nonspecific responses that are detrimental to their health and productivity [75]. It has been well established that transportation stress results in a reduction in production performance, increased morbidity and mortality [76,77].

#### 5.3.1. Handling of Goats after Transport

During the transportation of animals, handling is too critical to prevent exciting the animals as it requires up to 30 min for an animal to calm down after rough handling [78]. The animal handlers should move very slowly and deliberately without agitating the animals. While calm animals move easily, when they become agitated, it may be difficult to remove them from a pen [73,78]. Further, handlers should not force or push the animals to move with crowd gates as it creates difficulty in handling. Goats are social animals, and they should be handled in groups instead of individually. In addition, there should be proper ramps and platforms for smooth loading and unloading with cross slating (10 cm high × 30 cm deep) and 20 degrees slope to facilitate walking and prevent slipping [78,79].

#### 5.3.2. Preloading Precautions

Goats are highly socialized, and when they are unable to maintain visual contact with other animals for a long time, it causes emotional stress [80]. Therefore, it is essential to transport goats as a group with optimum spacing to avoid social stress. In addition, horned and hornless animals should not be mixed in the same vehicle as this may cause bruising and injury. Goats should also not be mixed with other species during transportation. Further, pregnant, diseased, injured and emaciated animals are unfit to be transported as they cannot withstand the transportation stress [79]. Goats should have free access to fresh water and feed both before and after transportation [81].

#### 5.3.3. Break Journey during Transport

Goats transported for 12 h over a distance of about 350 km exhibited significantly decreased live body weight compared to their preload weight [82]. Gupta et al. [75] also reported that transportation of goats up to 8 h during hot, humid and winter seasons causes stress, resulting in decreased body weight. The goats subjected to 7 h of road transport stress during hot, humid environmental conditions displayed oxidative stress and hematological derangements, which required 7 to 16 days to restore the normal condition [83]. In addition, goats are highly susceptible to respiratory infections after a long journey under adverse environmental conditions. The European Union regulations relating to the welfare of animal transport permit a maximum journey time of 19 h, which includes a 1 h break for feed, water, and rest every 8 to 9 h [84]. Goats should be offloaded after 24 h for feed, water, rest and exercise if the journey is to take longer than 30 h [79]. Therefore, it is essential to break the journey between 8 to 9 h for feeding and watering and to ensure the animals’ health status. Further, Chambers et al. [78] recommended a maximum distance of 24 km on the first day and 16 km on subsequent days for goats depending upon prevailing weather, body condition and age of the animal.

#### 5.3.4. Transport during Cool Hours of the Day

High environmental temperatures will increase the risk of heat stress and mortality during transportation [78]. It is preferable to transport goats during the cooler periods of the day: morning, evening, or even at night, particularly in tropical regions with high environmental temperatures [79]. This may reduce environmental stress and result in considerably more comfortable conditions for the animals during transportation. Further, the temperature within the vehicle must not go below 0 °C during a journey of more than 8 h. It is advisable to protect goats from heat, wind and cold by providing a proper cover over the vehicle during unfavorable weather conditions [80].

#### 5.3.5. Ensuring Clean Drinking Water Periodically during Transport

The transportation of ruminants for a long-extended period without feed and water results in loss of body weight [77]. The animal loses body weight due to the high-energy requirement for thermoregulation as well as respiratory and cutaneous water losses, particularly in summer [85]. The low water turnover rates of goats mean that they are better adapted to withstand dehydration than sheep under dry climatic conditions where goats drink water occasionally during transport [22]. However, withholding feed coupled with dehydration may lead to live-weight loss or shrinkage as high as 10% in the summer [80]. Hence, ensuring fresh and clean water is necessary during the transportation of goats to prevent dehydration. Lactating and young goats must be provided water every 8 h, while adult goats may need to be watered every 12 h during transport [79].

#### 5.3.6. Supplementation of Electrolyte during Transport

Handling, loading and transportation adversely affect the electrolyte balance of goats and results in respiratory alkalosis, dehydration and muscular damage [74]. The modified hormonal status activates the alteration in mineral profile during transport stress in combination with increased metabolism of calcium, magnesium, sodium, potassium and chloride [86]. The supplementation of vitamin C and electrolytes helps in reducing transportation stress in goats [81,87]. The oral electrolyte rehabilitation with similar interstitial fluid constituents sustains both live and carcass weight with improved meat quality in meat animals [75]. Supplementing goats with ascorbic acid can restore body weight and ameliorate the adverse impacts of multiple stresses of goats transported during the hot-dry seasons in tropical regions [88]. Administration of ascorbic acid 30 to 40 min prior to transportation alleviates the stresses caused by handling, loading, transportation and the concomitant effect of high ambient temperature and relative humidity on the erythrocytes of goats [89]. Gupta et al. [75] also reported that supplementation of vitamin C and electrolytes could be even more beneficial in ameliorating transportation stress in goats.

## 6. Effect of the Production Systems on Welfare of Goats

The importance of animal welfare in animal production systems is rising due to ethical concerns as well as economic costs. Good animal health and welfare are associated with the maintenance of physical, mental, social and ecological wellbeing and the freedom from diseases [90]. Goats are reared under different systems varying from intensive to pasture-based systems with various production protocols ranging from intensive milk production to extensive meat production. It is a prerequisite that animals should be free of thirst, hunger and disease for productivity and humanitarian considerations. The production and management systems can modify animal response and behavior, which may affect animal welfare [91].

Generally, goats are raised in three different farming systems, viz intensive, semi-intensive and extensive farming systems. However, in many parts of the world, predominantly extensive goat rearing is practiced in which animals are provided with fewer facilities like minimal/no health management practices while grazing on natural pastures [53,92]. In an extensive system, small ruminants may have compromised welfare due to nutritional stress, limited water supply, climatic extremes, parasitic infestation and lameness, culminating in low production and reproduction and a high mortality rate [92]. The productivity of goats in extensive systems mainly depends on climatic conditions, availability of feed and water and soil fertility [93]. The structure and composition of pasture influence grazing goats’ welfare and performance since it modifies the ingestion behavior, herbage intake, and production performance of goats. Goats in extensive production systems often face nutritional deficiencies that might affect the welfare, health and growth potentials. Consumption of vegetation containing toxic substances and anti-nutritional factors could cause a severe threat to the health and welfare of goats reared in the extensive system [94]. Further, goats possess the ability to browse a wide range of pasture due to their bipedal stance behavior. Further, goats also can consume succulent cactus and high fiber content in a stalk of a tree during drought conditions, which was in contrast to other species [22,25]. In addition, they also possess the ability to travel long distances, which imparts them the potential to search the limited pastures [22].

While goats are well adapted to harsh environmental conditions, they can also be reared in intensive systems with permanent housing. However, intensive production of dairy goats requires specialized husbandry practices. In the intensive farming system, goats are confined with a high density of animals per unit area, controlled feeding and some management practice, which may negatively influence their behavioral responses and production performance. Hence, the goats need appropriate spacing without overcrowding in the animal house, which protects them from the harsh environmental conditions [95]. The feeding systems should ensure that the nutrient requirements are met with an approach of supporting the natural ingestion behaviors of goats [96]. The milking management practices, such as adaption to machine milking, pre-parturition training to milking parlor and type of milking, may largely affect the welfare, health and production performance of dairy goats. The stress caused by fear of humans and handling has practical implications on dairy goat performance. Therefore, decreasing the emotional or physical stress of dairy goats may enhance their productivity and health status. However, over-milking, malfunction of machine milking systems and poor hygiene during the milking process may negatively influence the immune system and increase the risk of mastitis [97]. Hence, it is essential to maintain cleanliness in the housing systems, milking procedures and udder care with milker hygiene to ensure goat welfare and health in intensive production systems [93].

Water is an essential nutrient for all animals for the maintenance of all bodily systems and survival. It plays a major role in fluid balance, thermoregulation, electrolyte balance and satisfying thirst. Periods of water scarcity may affect the goats’ health and wellbeing since it is associated with reduction of feed intake, increased rectal temperature, respiratory frequency, hypoglycemia, and increased blood urea nitrogen [98,99].

Among the livestock species, goats are considered the best-adapted animals to thrive under extreme climatic conditions, especially to high ambient temperature [25]. In a semi-intensive system, animals are provided shelter, supplemental feed and health management practices. In an intensive system, goats are reared with the goals of higher productivity per animal or production per area available, cultivation and fertilization of pastures, division of pastures in paddocks and breeding season being a frequent management practiced in this system [93]. Goats reared in intensive systems face welfare issues because goats are stocked at high-density, with less resting area and possible accumulation of feces and urine.

Poor ventilation is another issue in intensive systems that can cause negative impacts on health, welfare and production performance by affecting the heat exchange between the animal and its environment, increased relative humidity and accumulation of noxious gases, dust and airborne microbes [100].

Repeated and inappropriate handling of goats by stockpersons in intensive systems may cause stress to the animals. Generally, goats exhibit ancestral predatory fear, difficulty in adaptation to unfamiliar environments and poor integration with unknown animals. Thus, goats suffer more greatly than other livestock species when any changes are made in housing and management practices like frequent regrouping, feeding and change in stockman. The introduction of new goats to a herd can increase aggressive behavior, disrupt the social structure of an established herd, and alter the goats’ social hierarchy [101]. Regrouping or moving of animals can also have a negative effect on goat welfare and production performance [102].

Generally, people mainly focus on the animal welfare issues in intensive systems, whereas animals under extensive systems attract less attention. Currently, extensive goat production systems are expanding worldwide [103]. An extensive goat production system provides the behavioral needs of the animals in their natural habitat, and animal movement and exercise may alleviate stress. The pastures also provide a comfortable lying area compared to the intensive system and prevent the occurrence of many diseases, particularly in dairy goats. The welfare of goats is preserved under extensive systems in a natural environment, and the goats thrive by efficiently using the natural resources. However, the uncertainty of the environmental variables in association with human management practices may not cater to all the needs of the goats, and welfare issues may arise in an extensive system [104]. Therefore, goats selected for an extensive production system should be adapted for the particular environmental conditions to perform well with few welfare problems [91]. While extensive goat production systems are not absolutely free from welfare issues, it is less compared to intensive systems. The advancement in management and husbandry practices, such as technological developments that facilitate remote supervision of the animals on pasture conditions, may reduce welfare issues in an extensive system.

## 7. Different Approaches and Methodologies to Assess Welfare in Goats

There are various approaches and methods by which the welfare of heat-stressed goats could be assessed. The particular section highlights various behavioral approaches and different methodologies to assess welfare in goats.

### 7.1. Different Approaches to Assess Welfare in Goats

#### 7.1.1. Naturalistic Approach

The naturalistic approach allows goats to be raised and assessed in their natural environment. This approach involves animals being studied in wild or semi-wild and comparing with captive animals. Since the goat has been domesticated for over 10,000 years [105], their natural behavior may have changed. However, their behavior can be compared with the behavior exhibited by the wild or feral goats. Providing such natural environmental conditions to ensure welfare, especially from the poor and marginal farmers’ perspectives, remains a challenge. However, suppose the natural environmental, ecological condition is ensured. In that case, the animals could exhibit normal behavior, imparting them the potential to maintain better health both mentally and physically, culminating in better economic returns to the farmers [106]. The goat is a herd animal and follows a hierarchical system, and thus, keeping them in a group may be beneficial instead of keeping them in isolation [107]. Further, the distance between the watering spot and the grazing area should be close enough such that the animals can visit the spot at least once a day and this was considered a naturalistic approach to ensure the welfare of the goat [22]. There are several indicators by which the welfare in goats could be assessed, including hair coat, abscesses, nasal discharges, oblivion, and queueing for feed. Further, animal-based indicators, such as lameness, lesions on the head, body, leg injuries, BCS, ocular discharges, behavior assessment and water availability, need to be considered [53].

Under extensive systems, a familiar human approach test needs to be implemented as goats do not come in frequent contact with humans [102]. This test evaluates the animal fear and determines the previous human–animal interaction. Thus animals’ reaction to the nearing humans is measured [65]. Indicators like the cleanliness of facilities also need to be incorporated in an extensive system as animals spend the night together in the same enclosure. Commonly faced compromises under extensive systems are inadequate water and feed, climate stress, parasitic disease and lameness [92]. Free-ranging in the extensive system helps the animals to exhibit their normal physiological and behavioral functions. However, extensive walking in search of fodder can exhibit stress compromising the welfare. Further nutritional stress during the mating season will worsen the welfare of goats [108]. In addition, lameness also poses a significant problem under extensive goat rearing systems [109]. The naturalistic approach favors good animal welfare as the goats are reared in a more natural environment, thus promoting healthy and quality animal products. However, negative impacts like fear, injury, hierarchal structures, rivals, predators, and environmental effects are some of the disadvantages of naturalistic approaches without compromising their natural behavior and physiology. Thus, in the natural approach, raising the goats in semi-intensive to free-range systems is preferred to minimize welfare issues. Providing the feed in the natural habitat and natural form and maintaining social contacts with the herd with reduced human contacts would be the ideal platform for the goats’ to exhibit their normal behavior.

#### 7.1.2. Functional Approach

Under a functional approach, growth and reproductive performance, physiological function and behavior that aid in countering the stress and helps in their longevity and biological fitness is monitored as a measure of welfare [110]. The importance is placed on the biological functions rather than the emotions and feelings of the animals. Research methods for function-based approaches include measuring growth, production, reproduction rates as well metabolic biomarkers. Other methods in the function-based approach are veterinary epidemiology methods and veterinary pathology methods. Measuring cell-mediated and humoral immunity and measuring stress level using plasma cortisol, non-esterified fatty acid (NEFA), heat shock protein 70 (HSP70) as non-invasive biomarkers along with their relationships with the physiological variables like respiratory rate, pulse rate forms an integral part of function-based welfare assessment [110]. Similarly, during heat stress, non-invasive and semi-invasive methods like the use of IR radiation for measuring body temperature and measurement of plasma enzymes can help in the welfare assessment of the heat-stressed goats based on a functional approach [111]. One such study in goats involving a functional approach based on direct observations identified lameness, overgrowth of claws and lesion of teat, udder, skin and purities as major concerns and indicators of poor welfare on 24 UK commercial dairy goat farms [112]. Similarly, lameness was a major concern in goats kept in 71 farms in Nigeria [113]. Goats in a herd in France exhibited 12.5% lameness [114], while 24% lameness was reported in dairy goats in another herd [115]. In the UK, 91.2% of farms had claw overgrowth problems due to failure of hoof wear when housed on straw bedding [116]. Likewise, udder and teat lesions affect the welfare and production of goats [117]. A study of 32 farms in northwestern Italy showed that proper cleaning procedures adopted during milking occurred in 86% and 83% of the intensive and semi-intensive farms, respectively. Similarly, the frequency with which animal bedding materials were changed influenced milk production and microbial counts on the floor. More frequent bedding changes decreased microbial count, improved udder health and increased milk production and the welfare of the goats. Hair coat, BCS, cleanness and severe lameness did not vary in between intensive and semi-intensive farms. Good management practices like antiparasitic treatment were attributed to good BCS conditions of goats. However, another study in goats showed BCS was not influenced by caseous lymphadenitis [117]. Other functional approaches of poor welfare and associated issues include pre-pathological symptoms like elevated plasma cortisol concentrations, increased body temperature, increased heart rate, excess neuro-endocrine function, suppressed immune system and suppressed fertility. Likewise, the pathological symptoms include chronic disease, self-mutilation behavior, injuries, behavioral issues, stereotypies and expression of fear. Post pathological symptoms include physical scars, health wounds and behavioral and disturbance injuries.

#### 7.1.3. Subjective Approach

A subjective approach to measuring welfare involves judging the welfare of the animal based on feelings and emotions. This approach utilizes the discipline of animal psychology to interpret and understand both the positive and negative feelings exhibited by the animal during both distress and wellbeing states. The negative feelings include pain, suffering, and discomfort, while the positive feelings are the basic needs, freedom, comfort and pleasure. Identification of clear emotional indicators is also one of the key indicators in animal welfare [118]. Isolation of goats from their social group increases plasma cortisol concentrations, which correlates with increased emotional stress [76]. Among the different approaches to study the feelings and emotions, discrete emotions can be used, which implements the fundamental emotions. The dimensional approach characterizes the emotions based on two aspects of arousal (excited vs. calm) and valence (happiness vs. sad). The use of the dimensional approach is promising for studying animal emotions [118]. The use of vocalization among goats to identify the emotions seems to be a promising emotional indicator [119]. The use of arousal indicators can minimize the stress during negative situations, while accurate valence allows differentiating the positive and negative situations, thus enhancing animal welfare by promoting positive emotions [119]. Weaning of kids at an early age has resulted in reduced growth and psychobiological disturbance [120]. Stereotypies and abnormal oral behaviors were observed in early-weaned kids [121]. Animal welfare increases when they predict the adverse situation. However, when they fail to predict the situation, this can lead to a more adverse situation resulting in chronic physiological stress syndrome. Inability to predict and adapt to adverse situations can manifest as apathy ending in poor welfare [121]. When the animal is alert, exhibits play behavior, displays no or minimal abnormal behavior, is confident, moves around freely, does not display fear, can rest in a relaxed manner without constant vigilance and exhibits less unpleasant feelings are the important criteria to identify the good psychological state with minimal unpleasant feeling to cope to the adverse environment [122]. For example, spraying water during heat stress reverses the adverse behavior displayed by goats, thus promoting better welfare [25]. Therefore, positive and negative emotions can help identify the distress calls and positive feelings, which can be a healthy indicator of a subjective approach in goats.

### 7.2. Different Methodologies to Assess Goat Welfare

Animal welfare assessment is considered one of the important pillars of productive, efficient and sustainable farm animal production systems. This demands the assessment of the welfare of animals at a farm level and developing species-specific welfare protocols for on-farm welfare assessment [123]. Animal welfare assessment is a multidimensional approach to evaluate multicriteria issues with the aim to determine the actual welfare of animals, including both their physical and mental health [94,124]. The main factors affecting the welfare of farm animals are the physical environment, resources available to the farm’s animals, and management practices. Animals adjust to these inputs with their behavioral and physiological means [65].

Generally, two types of methodologies are available to assess the welfare of the animals. These are (1) animal-based measures include behavioral measurements, health and production records, including disease symptoms and (2) resources and managementbased measures include stocking density, workforce, housing conditions and health plans [12]. The resource and management based measures generally take into account the inputs that could affect animal welfare, while animal-based measures take into account animals’ response to assess the welfare of animals at the farm level [65]. Such approaches provide a more precise welfare assessment because they give information about the responses of animals as well as the adverse impact of the microenvironment on the animal. Animal-based measures seem to be more appropriate for measuring the actual welfare state of the animals [53]. Because environmental assessment, i.e., resource-based and management-based indicators, should be considered as risk factors that may affect animal welfare. These indicators show diversity across the world due to different shelter and management conditions [124]. Further, both species, as well as breed differences, clearly exist while following a welfare assessment protocol [65].

Two welfare assessment protocols were developed with various animal-based indicators to assess the welfare of farm animals. These are (1) Welfare Quality^®^, specially designed for pigs, poultry, dairy and beef cattle; (2) animal welfare indicators (AWIN), developed for sheep, dairy goats, horses, donkeys and turkeys [65]. The Welfare Quality^®^ protocol was developed with four principles and twelve criteria of welfare; most of the criteria also presented in AWIN protocols [65]. Each criterion includes various animal-based welfare indicators that can be found in more than one principle. Several animal welfare indicators should be included in the efficient animal welfare assessment protocol since they are all important and cannot compensate for each other [123].

Animal-based measures can be divided into four categories, physiological, behavioral, health and record-based [125]. Body condition score (BCS) is performed to evaluate the goats’ nutritional and health status [11]. It is a widely used method across species to evaluate changes in body fat reserves. The BCS is also related to the amount of feed offered, quality of feed, chronic diseases or parasitic infestation, and chronic exposure to heat or cold stress. Therefore, BCS is another reliable method linked with health, welfare and production [125].

Hair coat condition is another good indicator of health status or presence of ecto or/and endoparasites [126]. Hair coat assessment can be performed in all external body parts except the head and extremities of the legs. Hair coat indicators should be validated by clinical examination of hair quality in goats.

Thermal comfort is another method to assess welfare, and although goats are considered as highly adapted animals, heat or cold stress could affect their health, welfare and production performance [125]. Thus, thermal comfort could serve as an important indicator for the welfare of goats reared, particularly in extensive systems. Similarly, a few physiological variables like water intake, rumination rate, rectal temperature, pulse and respiration rate [127], skin temperature [127], and blood variables, such as glucose, total cholesterol, urea, and cortisol [128], have been identified as good indicators of animal welfare in a tropical climate.

## 8. Different Indicators of Goat Welfare

### 8.1. Behavioral Indicators

Behavioral responses are among the most important early indicators of the welfare of farm animals and reflect the early response of an animal to its environment [129]. Animals’ welfare could be assessed by simple observation of animal behavior, integration of perceived details of behavior, posture and movements. The quality of animal behavior was evaluated using descriptors, such as tense, content, relaxed, agitated, playful, which could provide information directly related to animal welfare [130].

Generally, goats from hot climatic regions, such as tropical, subtropical, arid and semi-arid regions, are more tolerant of solar radiation, heat stress and feed and water scarcity [131]. During heat stress conditions, goats exhibit both behavioral and physiological changes to maintain their body temperature. Reduced feed intake, increased water intake and drinking frequency, changes in urinating frequency, defecation, standing time, lying time, and seeking shade behavior are the major behavioral changes in livestock species exposed to heat stress [12]. Shade-seeking behavior is one of the foremost behavioral responses exhibited by heat-stressed animals. However, indigenous animals in tropical regions are considered more heat tolerant, spending more time grazing than resting in the shade [2]. Reduced feed intake is another important behavioral indicator exhibited by heat-stressed animals to reduce metabolic heat production during hot conditions [2,90]. It is an adaptive mechanism exhibited by the animals during heat exposure since digestion of feed is an important source of heat production. Increased water intake and drinking frequency are essential for heat dissipation and maintaining milk secretion [90]. In a hot environment, goats spend more time standing to reorient themselves in different directions to avoid the impact of direct solar radiation and ground radiation [90]. A standing posture also facilitates heat loss from animal bodies to the surroundings by exposing the body surface to the wind flow [2]. Rumination time and urinating frequency also get reduced in goats exposed to heat stress, and this was considered an adaptive mechanism to cope with heat stress [132].

Goats generally express their social and species-specific behavior and interactions with humans. Social behavior is critical since goats are commonly housed in groups and allowing them to express normal, non-harmful social behaviors (e.g., allogrooming). Social behavior could be changed due to management practices (repeated regrouping), housing conditions (stocking density, resting area) and lack of feed and water resources. Expression of agonist behavior (expressed to achieve resources and to establish dominance) is considered as a negative expression of social behavior in cattle [133]. Agonistic behavior in the goat could be exhibited as aggression with contact (e.g., biting, bumping) or aggression without contact (e.g., chases, escapes, threat displays) [134].

Compared to other farm animals, goats have a high rate of aggressive interactions [135]. Agonistic behavior is considered a welfare indicator and is commonly related to the reduced size of resting area, regrouping and limited feed and water resources). Queuing at feeding may also be used to evaluate the quality of social interactions among goats [136,137]. These authors observed that goats that had a low rank in the hierarchy spent more time queuing than the medium and higher ranked goats, which revealed that these competitive environments are a welfare issue for subordinates.

Vocalizations may be an interesting indicator of welfare in goats, which increases in response to social isolations and could be interpreted either as an adaptive and active response to communicate with companions or as a sign of distress and fear [138]. Further, excessive scratching or rubbing and abnormal oral behavior can be considered promising indicators of poor welfare in goats [132]. Self-suckling and inter-suckling are also abnormal oral behaviors commonly observed in certain goat breeds [139].

### 8.2. Physical Indicators

Assessment of animal welfare necessitates several indicators that comprise several facets, such as health status, physical comfort, and wellbeing of physiological and behavioral responses [123]. The animal-based indicators are also essential to evaluate the heat stress in goats, such as skin temperature, BCS and hair coat conditions [127,129]. Generally, BCS was found to be decreased in heat-stressed goats [20], and this was attributed to the depletion of body reserves in heat-stressed animals to ensure a regular supply of energy to maintain homeostasis.

The hair coat condition is a good indicator of the goat’s nutritional and health status [130], where shiny hair reflects good health and scurfy hair indicates poor health [53]. In addition, the health status of a goat may be assessed by BCS, which reveals the muscle and fat deposits on a five-point scale (1 to 5) [71]. The BCS 4 or above indicate fatness or obesity, while scores lower than 2 are considered thin and emaciated animals [53,140]. The low BCS depends on an intense mobilization of body fat reserve due to reduced energy intake and higher energy requirements, which occur during heat stress conditions in goats [96,141]. The increase of body surface temperature is also an indication of heat stress in goats [54]. Higher body surface temperature is directly associated with skin capillary beds’ vasodilatation to increase the blood flow to facilitate the heat dissipation to animal surroundings [21].

The resource-based welfare indicators, such as environmental temperature, relative humidity, solar radiation, wind speed, and rainfall, are important elements of the atmosphere that can be monitored and recorded to evaluate animal welfare [35,128]. Such environmental factors influence the thermal comfort of goats [142]. The desirable environmental temperature for goats varies between 6 to 27 °C with 60 to 80% of relative humidity and wind velocity of 0.5 m/s. Further, the thermal adaptation intervals vary from comfortable (16 to 25 °C) to hard (25 to 35 °C) where the physiological mechanisms are initiated to defend against heat stress and upper critical temperatures (35 to 38 °C) in which the animal must combat with its maximum potential to restore the homeostasis [143]. The computation of temperature and relative humidity in a single value as temperature–humidity index (THI) has been adopted as a universal tool for evaluating animal welfare under heat stress conditions. The THI values of 70 or less are considered comfortable for goats, whereas they experience heat stress beyond 75. The THI above 77 potentially causes moderate heat stress, and above 85 is a condition of severe heat stress in dairy goats [21,63]. Further, goats compromise their homoeothermic capacity when the THI exceeds 80 [131].

### 8.3. Physiological Indicators

Physiological responses to heat stress include increased rectal temperature, respiration rate, heart rate and sweating to facilitate the activation of heat dissipation mechanisms [2]. The changes in rectal temperature, heart rate and respiration rate are the primary welfare indicators of heat stress in goats [72,144]. The body temperature is a prospective welfare indicator in homeothermic animals due to its consistency with small variations, and rectal temperature is considered a good index of body temperature. Therefore, rectal temperature is extensively used as a physiological means to monitor animal welfare during heat stress. As homeotherms, goats try to constantly maintain their core body temperature around 39 °C with small variations [126]. The increase in the body temperature is a normal phenomenon by which animals can transfer the heat load from the body core to the periphery during heat stress [145]. An increase of 1 °C or less in rectal temperature tends to affect the welfare and performance of the goat during heat stress conditions [146].

The increased respiration rate in goats during heat stress is directly associated with the enhanced evaporative cooling mechanisms to restore the core temperature and meet the higher demand for oxygen [54,72]. Hence, the respiratory frequency could be an excellent welfare indicator to quantify the degree of heat stress in goats [21]. The respiration rate is an efficient and reliable measure of heat stress, which varies from 15 to 30 breaths/min in goats under thermo-neutral conditions [22,147]. The respiration rate is directly influenced by the immediate microenvironmental conditions and increases with the increase in environmental temperature to facilitate the evaporative heat loss in heat-stressed goats. Thus, respiration rate increases by 40–60 (breaths/min) when heat stress is low and increases by a further 60–80 (breaths/min) during moderate heat stress [22]. The respiratory rate accelerates when the heat load further high by 80–120 (breaths/min), and it goes beyond 150 breaths/min as the heat stress becomes very severe [21,22].

The respiratory frequency is a highly consistent parameter that helps assess heat load or severity of heat stress in goats. The panting scores are assigned based on the visual observations of respiratory patterns of goats using a 5-point [127] or 3-point scale [143]. The 3-point scoring system is very simple and adequate to distinguish different heat stress levels as goats are respiring with closed mouth with slight flank movements designed ‘score 0′ as normal [143]. Score 1 describes the varying signs of the accelerated respiratory frequency with closed mouth panting, while score 2 reflects heavily to severe open mouth panting with protrusion of tongue and excessive salivation [63,143].

The heart rate is the regular beat rate of the arteries as the blood is pumped through to the heart [148]. The heart rate varies from 60 to 80 beats/min in goats at resting conditions [24,49]. The exposure of the goat to heat stress increases the heart rate from 74 to 91 (beats/min), and the increase may be due to elevated respiratory, muscular activity and decreased vascular resistance to blood flow at the periphery [49,146]. Further, the increase in heart rate during heat stress facilitates higher heat loss by the enhanced blood flow from the core to the peripheries of the body [111,144,145]. Thus, the heart rate could be considered another important reliable welfare indicator during exposure to heat stress in goats.

### 8.4. Productive Indicators

Animal welfare is highly essential to maintain a conducive environment where the animal may perform its maximum to achieve optimum productivity [149]. Heat stress is one of the major factors that negatively influence domestic animals’ health and welfare status and productivity. In general, goats are well adapted to a wide range of climatic conditions, and particularly the indigenous breeds perform efficiently in their native environments. However, the high producing dairy goats react negatively to high environmental temperatures, and specifically, the purebreds are very susceptible to heat stress [57]. The hot environmental temperatures reduce the growth and milk production with impaired reproductive performance. Heat-stressed goats compromise their growth to support life-sustaining activities [66]. The reduction in growth rate is evidenced by the downregulation of growth-related gene expression in heat-stressed goats [71]. Heat stress reduces milk production by 9% in early lactating dairy goats with greater reductions in milk fat and protein percentage with a higher level of somatic cells [48]. The goat produces milk normally up to the THI level of 80, and the yield moderately decreases when THI reaches between 80 to 85 [150,151]. The milk yield is severely affected by heat stress as soon as the THI reaches 85 to 90 [37]. The decrease in milk yield and growth rate in goats during heat stress may be due to reduced feed intake and the process of metabolic adaptations [152]. Goats are extremely good in reproductive performance with higher fertility and short generation intervals [37]. However, heat stress affects the goats’ reproductive efficiency when the THI reaches above 70 in the mountain regions [37]. Further, Amitha et al. [153] reported that heat stress adversely affects the reproductive efficiency in goats by downregulating most of the reproduction-related genes’ expression patterns. The elevated environmental temperature affects reproductive performance by decreasing estrous expression and altered follicular growth with impaired embryonic development [154]. Thus, growth, milk production and reproductive variables could serve as reliable indicators for reflecting welfare in heat-stressed goats.

## 9. Conclusions

Heat stress is one of the major factors hindering livestock production as it compromises both production and welfare in goats. While goats adapt effectively to heat stress, in doing so, their production is compromised. Therefore, quantifying the various productive and adaptive responses may yield important indicators to reflect both production and welfare in goats. The commonly used indicators to quantify goat welfare during heat stress exposure are agonistic behavior, vocalization, skin temperature, BCS, hair coat conditions, rectal temperature, respiration rate, heart rate, sweating, reduced growth, reduced milk production and reduced reproductive efficiency. However, the set of indicators for goat welfare could differ according to the type of production systems in which they are reared. The most noteworthy indicators emerge from the extensive rearing systems. Therefore, a systematic approach is essential to quantify welfare response in goats based on how they perform in their natural environment, their growth and reproductive performance, physiological function, behavior, feelings, and emotions involving animal psychology. Such an approach may help to assess the stress level in goats during heat stress and probably may provide helpful information to implement appropriate strategies to ensure welfare.

## 10. Future Perspectives

More research efforts are needed to study goat behavior in their natural environmental condition using advanced facilities to understand the hidden intricacies of their welfare in depth. Welfare information may differ from breed to breed, and such information is very scarce. Therefore, efforts are also equally needed to screen indigenous breeds by subjecting them to various welfare assessment protocols. Such an action would help us understand the genotype–environment interaction and probably help the farming community identify agroecological zone-specific goat breeds. Such an approach would help the poor and marginal farmers to choose the most suited breed to ensure their economic return. The technological advancement needs to be explored to identify more reliable biomarkers for welfare in goats. Thus, genetic approaches, beginning from identifying heat-stress-associated genetic markers, validating their potential, and finally incorporating these candidate genes for future marker-assisted selection, could pave the way towards developing heat-tolerant goat breeds. Such superior breeds, having the advantage to withstand climatic extremes, could promise sustained production, thereby ensuring the livelihoods and economic returns to the farmers amidst the predicted climate change scenario. This could help policymakers develop suitable breeding programs using such biomarkers to develop new breeds with the potential for surviving and producing optimally in a given location. This could be the way forward to optimize goat production and to make goat rearing a profitable enterprise.

## Data Availability

Not applicable.
